# A comparative experimental study on the cross-plane thermal conductivities of nano-constructed Sb_2_Te_3_/(Cu, Ag, Au, Pt) thermoelectric multilayer thin films

**DOI:** 10.1186/s40580-018-0154-1

**Published:** 2018-08-08

**Authors:** Gang Yang, Jiahui Pan, Xuecheng Fu, Zhiyu Hu, Ying Wang, Zhimao Wu, Erzhen Mu, Xue-Jun Yan, Ming-Hui Lu

**Affiliations:** 1 National Key Laboratory of Science and Technology on Micro/Nano Fabrication, Shanghai Jiao Tong University, Shanghai, 200240 China; 2 Institute of NanoMicroEnergy, Shanghai Jiao Tong University, Shanghai, 200240 China; 3 Department of Micro/Nano Electronics, Shanghai Jiao Tong University, Shanghai, 200240 China; 4 Center for Advanced Electronic Materials and Devices (AEMD), Shanghai Jiao Tong University, Shanghai, 200240 China; 5 National Laboratory of Solid State Microstructure & Department of Materials Science and Engineering, College of Engineering and Applied Science, Nanjing University, Nanjing, 210093 China; 6 Collaborative Innovation Center of Advanced Microstructures, Nanjing University, Nanjing, 210093 China

**Keywords:** Thermoelectric thin films, Cross-plane thermal conductivity, Time-domain thermoreflectance (TDTR), Electron–phonon coupling

## Abstract

Thermoelectric multilayer thin films used in nanoscale energy conversion have been receiving increasing attention in both academic research and industrial applications. Thermal transport across multilayer interface plays a key role in improving thermoelectric conversion efficiency. In this study, the cross-plane thermal conductivities of nano-constructed Sb_2_Te_3_/(Cu, Ag, Au, Pt) thermoelectric multilayer thin films have been measured using time-domain thermoreflectance method. The interface morphology features of multilayer thin film samples were characterized by using scanning and transmission electron microscopes. The effects of interface microstructure on the cross-plane thermal conductivities of the multilayer thin films have been extensively examined and the thermal transfer mechanism has been explored. The results indicated that electron–phonon coupling occurred at the semiconductor/metal interface that strongly affected the cross-plane thermal conductivity. By appropriately optimizing the period thickness of the metal layer, the cross-plane thermal conductivity can be effectively reduced, thereby improving the thermoelectric conversion efficiency. This work presents both experimental and theoretical understanding of the thermal transport properties of Sb_2_Te_3_/metal multilayer thin film junctions with important implications for exploring a novel approach to improving the thermoelectric conversion efficiency.

## Introduction

With the ever increasing of energy demanded, to develop sustainable energy sources strategy becomes more and more concerned in recent years, Thermoelectric technology, harvesting electric power from heat, is a promising environmentally friendly means of energy conversion. The thermoelectric technology has an inherent advantage to harvest widely distributed waste heat, and also proved as an alternative route to convert solar energy into electric power economically [1]. In addition, the thermoelectric system also has other unique advantages such as small size, high reliability, no pollutants, and feasibility over a wide temperature range [2]. However, the current conversion efficiency of thermoelectricity is much less than that of equivalent mechanical systems. For this reason, thermoelectricity has been long limited to engineering or commercial application. Therefore, improving the conversion efficiency of thermoelectric devices is one of the most important challenges in the fields of energy harvesting.

The thermoelectric energy conversion efficiency is determined by the dimensionless figure of merit ZT, where Z is a measure of a material’s thermoelectric properties and T is absolute temperature. The materials coefficient Z is expressed in term of Seebeck coefficient S, electrical conductivity σ and thermal conductivity κ, yielding equation Z = S^2^·σ/κ. To maximize the ZT value of thermoelectric device, a large Seebeck coefficient, high electrical conductivity, and low thermal conductivity are therefore most expected [3, 4]. In order to improve the thermoelectric efficiency, various approaches to enhancing ZT have been proposed and developed.

In the early 1990s, Hicks and Dresselhaus pointed out that low-dimensional thermoelectric materials exhibit a significant enhancement in ZT value [5–7]. The enhancement mechanism can be attributed to two facts, one involving the increase in the power factor (S^2^·σ) by quantum confinement effects, which enhanced the density of states near the Fermi energy [8–10], and the other involving the reduction in thermal conductivity by tailoring the nanostructure [11]. The idea of selectively modifying thermoelectric material properties using lower-dimensional nanostructures has been successful developed in the subsequent years [12, 13].

Nanostructured thermoelectric materials offer a new way of improving thermoelectric conversion performance by tailoring electron and phonon transport properties [14, 15]. Two-dimensional (2D) multilayer thin films or superlattice structures can significantly enhance the ZT values of thermoelectric materials since electrons are confined to move in two dimensions and phonons can be effectively scattered by the high density of interfaces, which results in a decrease in the lattice thermal conductivity [16, 17]. Abundant experimental and theoretical studies have indicated that nanoscale multilayer thin films or superlattice have lower thermal conductivities than their conventional bulk materials as a result of size and interface effects [18–21]. Of special note, the traditional phenomenological heat transfer equations are not applicable at the nanoscale; the investigation or analysis of the thermal transport mechanism in the multilayer thin film system belongs to the catalog of micro/nanoscale heat transfer [22]. Scientific understanding of the thermal transport occurring in the nanoscale multilayer thin structures is highly necessary and exigent [23, 24]. Recently, the thermal conductivities of periodic thin film structures have been extensively investigated by Hopkins et al. [25–29], Xiao et al. [30], and Cahill et al. [31]. The results indicated that the transport of electrons and phonons in nanoscale multilayer thermoelectric thin films is mainly affected by the presence of interfaces and defects, and that the minimum thermal conductivity of a multilayer thin film or superlattice can be realized by optimizing its nanostructure [32–34]. In a series of papers, Chen et al. reported on their investigations of the ballistic-phonon transport occurring in the cross-plane direction of superlattices [34–36] that revealed the heat conduction mechanisms in the periodic thin-film structures. Hopkins et al. studied the effects of interfacial properties (roughness, disorder, dislocations, and bonding) on the cross-plane thermal conductivity of a multilayer thin film structure [25, 26, 29], and indicated that the cross-plane thermal conductivity can be further reduced by controlling the interface morphology [27]. However, the interfacial properties of multilayer thin films are closely related to the pair of the composed materials. Several studies indicated that ultralow thermal conductivities of the multilayers can be realized by choosing the pair of thermoelectric materials properly [37–39].

In this investigation, the cross-plane thermal conductivities of nanostructured Sb_2_Te_3_/(Cu, Ag, Au, Pt) thermoelectric multilayer thin films have been experimentally studied. The thermal transfer mechanism (i.e., size and interface effects) has been explored in combination with experimental measurements and modal analyses. The total effects of multilayer nanostructure on the cross-plane thermal conductivity of the thermoelectric thin film has been evaluated according to the obtained results, with the ultimate goal being to provide useful guidance and help for the practical thermoelectric engineering applications.

## Sample preparation

Low-dimension thermoelectric multilayer thin film preparation processes are generally categorized into two groups: physical and chemical processes, each having its own advantages and disadvantages. The choice of method mainly depends on the cost of processing or material properties. In general, physical methods include magnetron sputtering, electron beam physical vapor deposition, and molecular beam epitaxy, which inherently need a high-vacuum environment. The chemical processes include electrochemical deposition and metal-organic chemical vapor deposition, as well as other synthesis methods. These methods are more suitable for large-area deposition. In most cases, thermoelectric multilayer thin films are fabricated by physical methods depending on their properties and the applications. Among the various physical approaches, magnetron sputtering is commonly used. Magnetron sputtering has advantages compared to most thermal evaporation techniques: (i) the composition of the sputtered material is the same as that of the target, resulting in higher quality of the samples; (ii) the evaporation conditions are stable and easily controlled by plasma current; (iii) the heat load on the chamber walls is far smaller, reducing outgassing and subsequent impurity incorporation by reactive particles [40].

In this experiment, the bilayer thin films were prepared by alternate sputtering onto a Si substrate in a high-vacuum magnetron sputtering system (Denton, explorer-14, USA) at room temperature. The independent metal targets have high purity (99.999%). Before the process, the Si substrate was soaked in B.O.E solution (HF: NH_4_F = 1:6) for 5–10 min to chemically etch off the surface oxide layer, and then, was ultrasonically cleaned in acetone and ethanol solutions for 10 min. After drying with high purity nitrogen gas, the substrate was transferred into a sputtering chamber and mounted on the holder. The distance between the targets and substrate was about 200 mm. The sputtering pressure was set at 3 mTorr and the substrate pressure was controlled at 2 × 10^−7^ Torr. A calibrated quartz crystal monitor was used to measure the deposition rate. During the deposition, the power was applied in radio frequency (RF) mode for Sb_2_Te_3_ and direct current (DC) mode for Au, Ag, Cu, and Pt. In addition, the substrate was rotated at the speed of 20 rpm to ensure deposition uniformity. The total thickness of the thermoelectric multilayer thin film was about 300 nm.

## Multilayer structure characterization and analysis

The multilayer thin structure is strongly correlated to its thermal and physical properties. Particularly, the size and interface effects on the cross-plane thermal conductivity need to be further investigated. In order to explore comprehensively the interface effects on the thermal transport properties of the multilayer thin film, the cross-section morphology was observed by scanning electronic microscopy (SEM) and using a transmission electron microscope (TEM).

### SEM

**Fig. 1:**
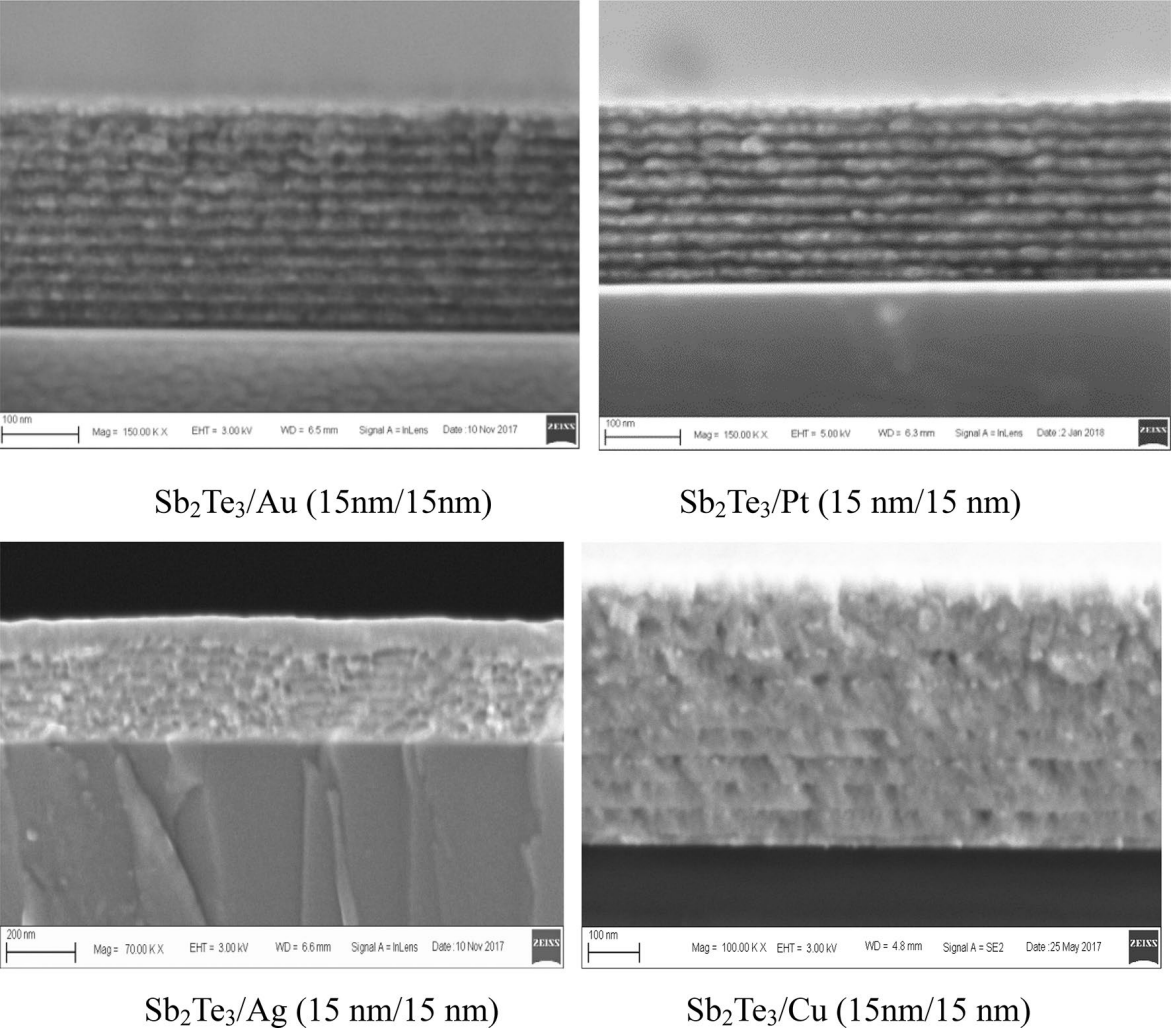
Comparison of the cross plane morphology of multilayer thin films with the same period thickness

**Fig. 2:**
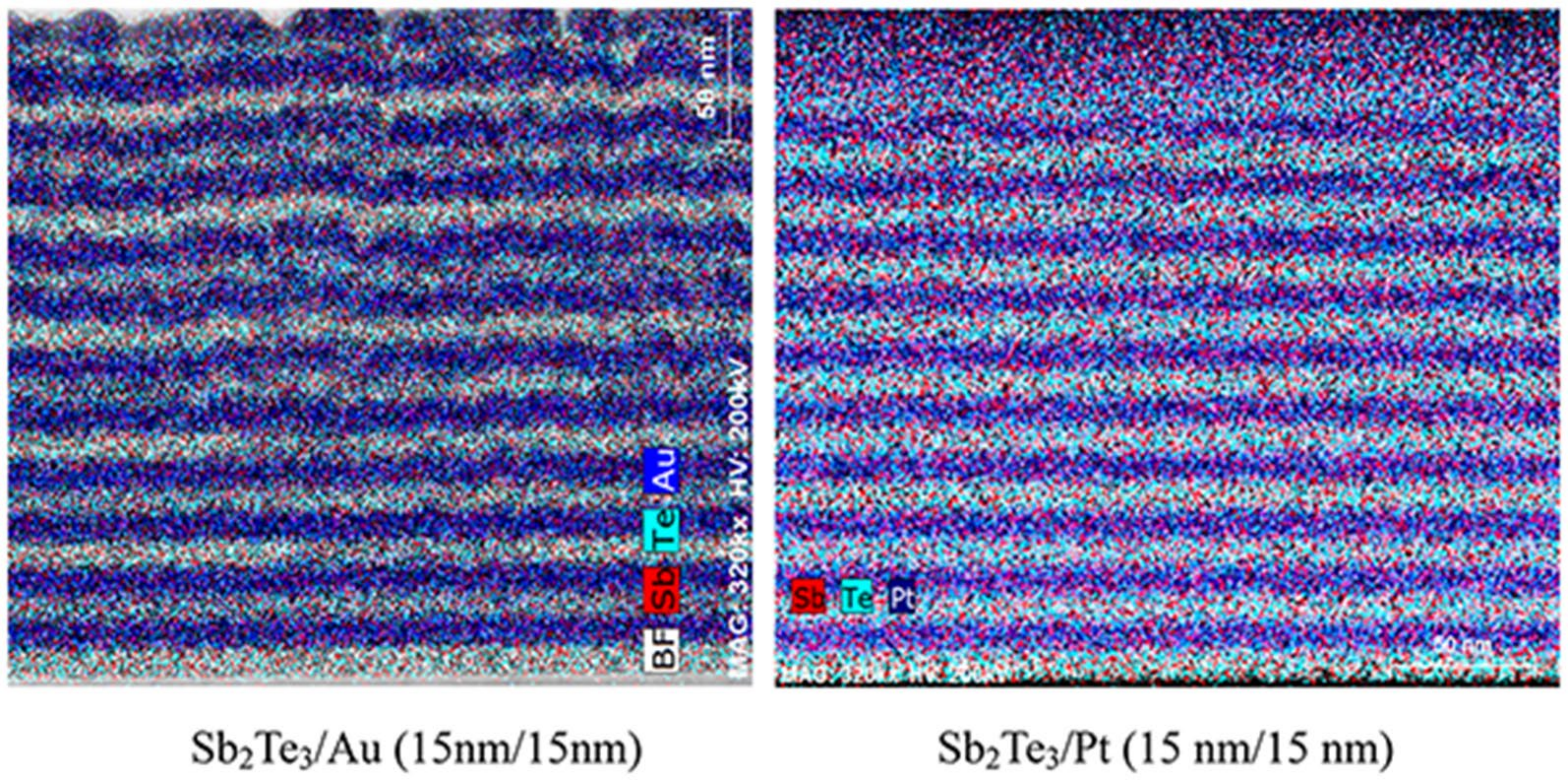
EDS images of Sb_2_Te_3_/(Au, Pt) thermoelectric multilayer thin films

The cross-plane morphology of the nano-constructed Sb_2_Te_3_/(Cu, Ag, Au, Pt) thermoelectric multilayer thin films were observed using a Zeiss ULTRA55-36-69 SEM. The images recorded at several resolutions were shown in Fig. 1. It can be seen from Fig. 1 that the grown multilayer films have a periodic structure consisting of alternating thin film layers. In order to examine the uniformity of period thickness, the multilayer thin film samples were also characterized by EDS line-scanning, and the obtained cross-section images are shown in Fig. 2. It can be seen from Figs. 1 and 2 that the typical layered structure is mainly parallel to the substrate surface; at a higher resolution, it is observed that the typical layered structure exhibits nanoscale roughness along the horizontal direction.

### TEM

**Fig. 3:**
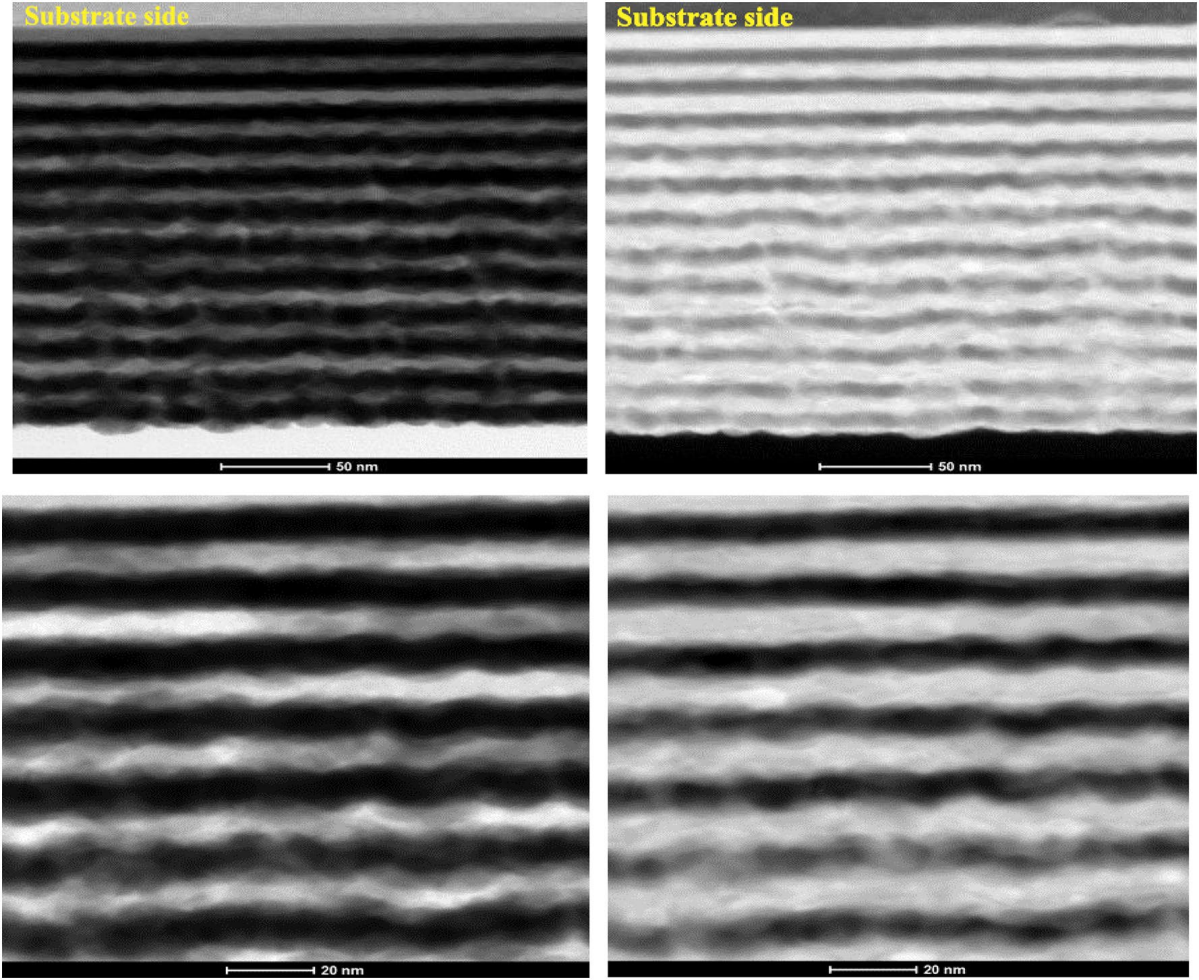
TEM cross-sectional images of Sb_2_Te_3_/Au multilayer thin films at different resolutions

In order to explore the microstructural features of the thermoelectric multilayer thin film systems, the alternate-layered structures were also observed using TEM (FEI, Talos F200X, USA). For convenience of observation, focused ion beams (FIBs) were employed to prepare the cross-section of the specimen. The TEM cross-sectional images of the Sb_2_Te_3_/Au multilayer thin films recorded at several resolutions are shown in Fig. 3.

Figure 3 shows the interface roughness between the Sb_2_Te_3_ and metal period layers. The interface roughness can affect phonon reflection and transmission at the interface; therefore, it can have a significant effect on the cross-plane thermal conductivity of the multilayer thin film. On the other hand, atomic scale interface roughness does not cause a significant reduction in electron mobility, since the typical electron coherence length in semiconductors is much longer than that of phonons, and it can be reflected specularly [18]. Hence, by constructing nanoscale Sb_2_Te_3_/metal multilayer thin film properly, it can reduce the cross-plane thermal conductivity without deterioration in electron transfer, thus improving the thermoelectric conversion efficiency. In practical engineering, the interface roughness correlated with certain sputtering parameters, such as Ar pressure, bias voltage, sputtering power and substrate temperature. It can be effective controlled by adjusted the bias power during fabrication.

**Fig. 4:**
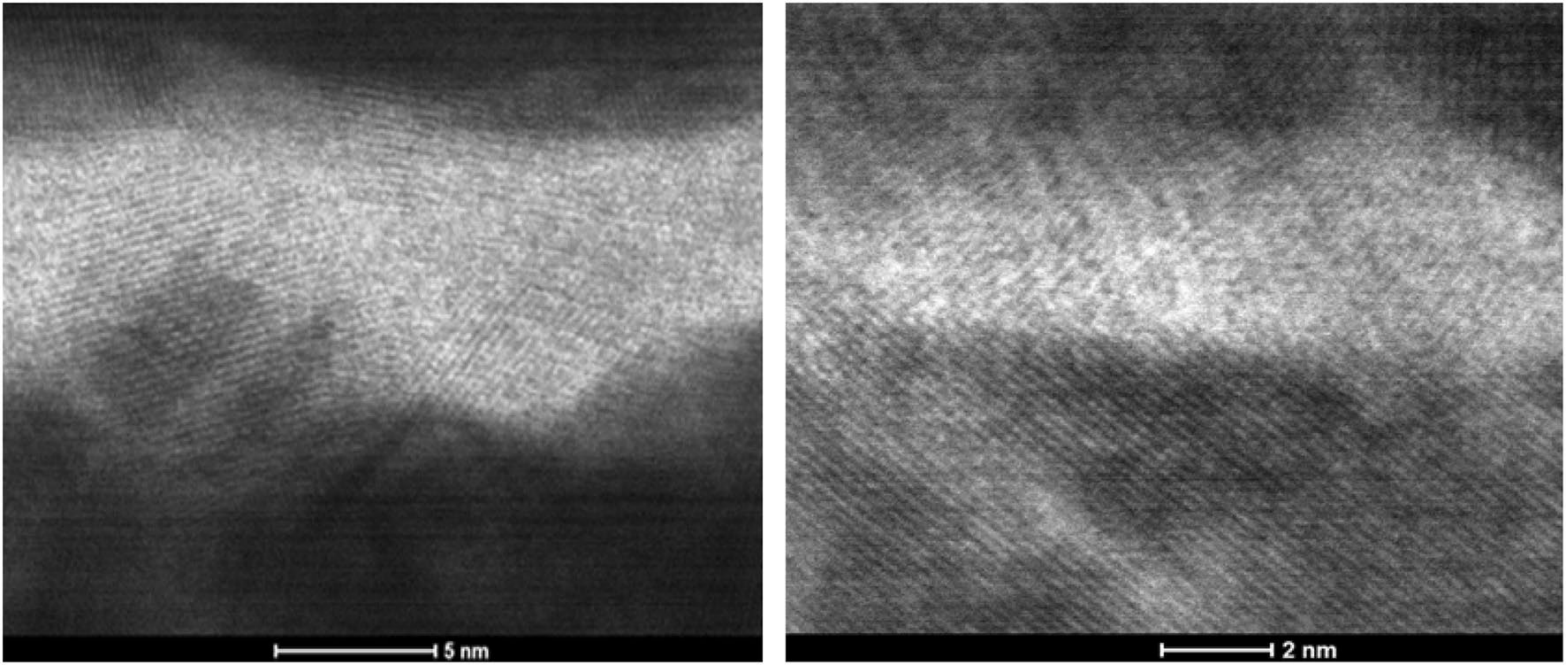
Interface microstructural features of Sb_2_Te_3_/Au multilayer thin films at the atomic scale

In order to explore the effects of interface morphology on the cross-plane thermal conductivity of Sb_2_Te_3_/metal (Cu, Ag, Au and Pt) multilayer thin films, the atomic-scale structural features of the interface were observed with a TEM at a higher resolution. The sample selected was 5 nm Au layer and the obtained cross-sectional TEM images of the Sb_2_Te_3_/Au layer at the atomic scale are shown in Fig. 4.

The atomic scale lattice structures of Sb_2_Te_3_ and Au at the interface can be clearly observed in Fig. 4. It shows the lattice dislocations at the Sb_2_Te_3_-metal layer interface. In fact, the lattice dislocations between the Sb_2_Te_3_ and metal in the multilayer structure can lead to a remarkable difference in the thermal and physical properties of the thin film systems. In addition, it probably had an influence on phonon transport. The lattice-dislocations interface with defects and mixing may affect the phonon transmission and significantly change the cross-plane thermal conductivity. To some extent, lattice dislocations or misfits at the multilayer interface can enhance phonon scattering, thereby reducing the thermal conductivity of the lattice [41]. Interfacial dislocations can enhance phonon or electron scattering, thereby reducing the lattice or electron thermal conductivity.

## Experimental procedure

In this experiment, the cross-plane thermal conductivities of nano-constructed Sb_2_Te_3_/(Cu, Ag, Au, Pt) thermoelectric multilayer thin film samples were measured by time-domain thermoreflectance (TDTR) method. The measurements were carried out in the National Laboratory of Solid State Microstructures of Nanjing University. Currently, TDTR was developed as a reliable and convenient non-contact technique for determining the thermal conductivities of nanostructured materials. Before the TDTR measurements, a compact Al film with a thickness of 80 nm was deposited on the thin film surface to serve as an optical transducer. The output of a Ti: sapphire oscillator laser was split into a pump beam and a probe beam with a relative delay time of 0–4000 ps. The modulated pump beam heated the Al surface periodically, creating a temperature fluctuation at 9.8 MHz. This fluctuation was then detected by the probe beam due to the linear temperature dependence of optical reflectivity on the Al surface and was recorded by a lock-in amplifier. The cross-plane thermal conductivity was obtained by fitting the measurement signals to a diffusive thermal model. In this experiment, every measurement was repeated three times, and the average value was obtained. In addition, we strictly checked the experimental errors before and after each measurement. More details about the measurement, data analysis, and modeling can be found elsewhere [42–44]. In addition, to verify the measurement data, we also employed the standard differential 3ω method to measure and compare the obtained results with those obtained from the TDTR method.

## Results and discussion

### Cross-plane thermal conductivities of Sb_2_Te_3_/(Cu, Ag, Au, Pt)multilayer thin films with various period thickness

**Fig. 5:**
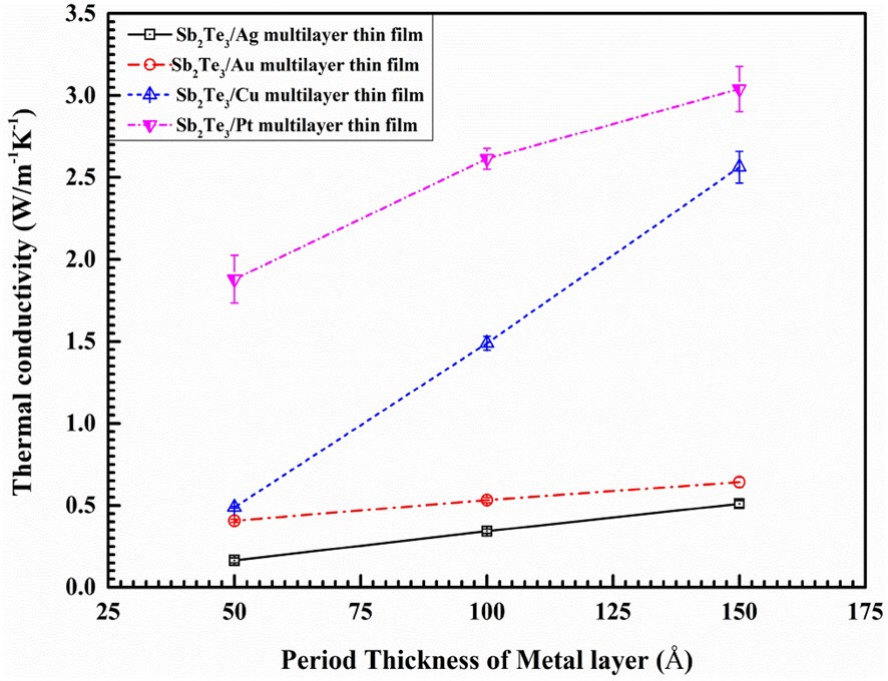
Cross-plane thermal conductivities of Sb_2_Te_3_/(Au, Ag, Cu, Pt) multilayer thin films

In order to investigate the effects of metal layer on traditional P-Type thermoelectric materials, Sb_2_Te_3_/X (X = Cu, Ag, Au, Pt) thermoelectric multilayer thin film with various period thickness were prepared using the magnetron sputtering method. The period thickness of the Sb_2_Te_3_ layer was fixed at 15 nm, the metal layer thickness (Cu, Ag, Au, and Pt) varied from 5 to 10 to 15 nm. The total thickness of the multilayer thin film samples was 300 nm. The numbers of period interfaces were 15, 12, and 10. The measured cross-plane thermal conductivities for the various samples are presented in Fig. 5.

It can be seen from Fig. 5 that the cross-plane thermal conductivities of Sb_2_Te_3_/(Cu, Ag, Au, Pt) multilayer thin films increase with the period thickness in a specific range (larger than 200 Å). The reason can be attributed to two aspects, one involving the increased proportion of the metal composition, and the other involving the reduction in the density of the interface. However, for the same period thickness, it can be found from Fig. 5 that the cross-plane thermal conductivity of Sb_2_Te_3_/Ag exhibits the minimum value compared to those of the Sb_2_Te_3_/(Cu, Au, Pt) thermoelectric multilayer thin films. In contrast, Sb_2_Te_3_/Pt reveals a high cross-plane thermal conductivity in all the samples investigated. There are two possible reasons responsible for the deviation of thermal conductivity of Sb_2_Te_3_/Ag and Sb_2_Te_3_/Pt multilayer film. One is the interface condition; it can be seen from the Fig. 1 that Sb_2_Te_3_/Pt film has good film forming characteristics than Sb_2_Te_3_/Ag film. The larger interface roughness of Sb_2_Te_3_/Ag multilayer thin film has a significant effect on phonon scattering, thereby reducing the thermal conductivity. Another reason is electron–phonon coupling occurred at interface of various materials pairs.

In a nonmetal–metal interface, there are two possible coupling pathways: one is the direct coupling between the electrons of the metal and the phonons of the nonmetal, and the other is the coupling between the electrons and phonons within the metal, and subsequently coupling the phonons of the metal and phonons of the nonmetal [45]. In theory, the coupling mechanisms are mostly studied using the two-temperature model, in steady state conditions, the two-temperature model assumed that the diffusive Fourier law of heat conduction is valid for both the electron and phonon gases separately, and the corresponding equations are given by

1 $$\kappa_{e} \frac{{d^{2} T_{e} \left( x \right)}}{{dx^{2} }} - G\left[ {T_{e} \left( x \right) - T_{p} \left( x \right)} \right] = 0$$

2 $$\kappa_{p} \frac{{d^{2} T_{p} \left( x \right)}}{{dx^{2} }} - G\left[ {T_{e} \left( x \right) - T_{p} \left( x \right)} \right] = 0$$

3 $$\frac{{d^{2} T\left( x \right)}}{{dx^{2} }} = 0$$

where *k*_*e*_ and *k*_*p*_ are the electron and phonon thermal conductivities, respectively, *T*_*e*_ and *T*_*p*_ are the electron and phonon temperatures on the metal side, respectively, *T* is the temperature on the non-metal side, and *G* is the electron–phonon coupling factor. The correlation between *T*_*e*_ and *T*_*p*_ can be obtained by subtracting Eq. (2) from Eq. (1) and integrating the resulting equation:

4 $$T_{e} \left( x \right) - T_{p} \left( x \right) = A\sinh \frac{x}{\xi } + B\cosh \frac{x}{\xi }$$

where *ξ* is the electron and phonon coupling length on the metal side, and can be given as:

5 $$\xi { = }\sqrt {\frac{{\kappa_{e} \kappa_{p} }}{{G\left( {\kappa_{e} + \kappa_{p} } \right)}}}$$

Combining Eqs. (1), (2), and (4), the temperature distributions of electrons and phonons can be obtained as:

6 $$T_{e} \left( x \right) = C + D\left( x \right) + \frac{{\kappa_{p} }}{{\kappa_{e} + \kappa_{p} }}\left[ {A\sinh \frac{x}{\xi } + B\cosh \frac{x}{\xi }} \right]$$

7 $$T_{p} \left( x \right) = C + D\left( x \right) + \frac{{\kappa_{e} }}{{\kappa_{e} + \kappa_{p} }}\left[ {A\sinh \frac{x}{\xi } + B\cosh \frac{x}{\xi }} \right]$$

Then, with the given boundary conditions, we can solve these equations and obtain the effective thermal conductivity for the bilayer thin film systems:

8 $$\lambda_{i} = \frac{{d_{1} + d_{2} }}{{\left[ {{{d_{1} } \mathord{\left/ {\vphantom {{d_{1} } {\left( {\kappa_{e} + \kappa_{p} } \right)}}} \right. \kern-0pt} {\left( {\kappa_{e} + \kappa_{p} } \right)}} + {{d_{2} } \mathord{\left/ {\vphantom {{d_{2} } {\kappa_{2} }}} \right. \kern-0pt} {\kappa_{2} }} + R_{i} + {{\left( {\kappa_{e} \xi \tanh \frac{{d_{1} }}{\xi }} \right)} \mathord{\left/ {\vphantom {{\left( {\kappa_{e} \xi \tanh \frac{{d_{1} }}{\xi }} \right)} {\left( {\kappa_{e} \kappa_{p} + \kappa_{p}^{2} } \right)}}} \right. \kern-0pt} {\left( {\kappa_{e} \kappa_{p} + \kappa_{p}^{2} } \right)}}} \right]}}$$

where *d*_1_ and *d*_2_ are the metal and nonmetal period thicknesses, respectively, and *R*_*i*_ is the phonon–phonon interfacial thermal resistance. Considering the multilayer thin film structure, Eq. (8) can be written as

9 $$\frac{{D_{N} }}{{\lambda_{e} }} = \frac{N + 1}{2} \cdot \frac{{d_{1} }}{{\kappa_{e} + \kappa_{p} }} + \frac{N - 1}{2} \cdot \frac{{d_{2} }}{{\kappa_{2} }} + \sum\limits_{n = 1}^{{N{ - }1}} {R_{n,n + 1} } ,$$

where *D*_*N*_ and *R*_*n*,*n*+1_ can be expressed as

10 $$D_{N} = \frac{{\left( {N + 1} \right)d_{1} + \left( {N - 1} \right)d_{2} }}{2}$$

11 $$R_{n,n + 1} = r_{n,n + 1} + \frac{{\kappa_{e} }}{{\left( {\kappa_{p} + \kappa_{e} } \right)}}\left\{ \begin{aligned} \frac{\xi }{{\kappa_{p} }}\tanh \frac{{d_{1} }}{\xi },n = 1,N - 1 \hfill \\ \frac{2\xi }{{\kappa_{p} }}\tanh \frac{{d_{1} }}{2\xi },n \ne 1,N - 1 \hfill \\ \end{aligned} \right.$$

Finally, as *N* increased, the effective thermal conductivity of the multilayer thin film can be expressed as

12 $$\lambda_{e} = \frac{{d_{1} + d_{2} }}{{\left[ {{{d_{1} } \mathord{\left/ {\vphantom {{d_{1} } {\left( {\kappa_{e} + \kappa_{p} } \right)}}} \right. \kern-0pt} {\left( {\kappa_{e} + \kappa_{p} } \right)}} + {{d_{2} } \mathord{\left/ {\vphantom {{d_{2} } {\kappa_{2} }}} \right. \kern-0pt} {\kappa_{2} }} + 2R + 4\frac{{\kappa_{e} }}{{\kappa_{p} }}\frac{\xi }{{\kappa_{e} + \kappa_{p} }}\tanh \frac{{d_{1} }}{2\xi }} \right]}} .$$

**Fig. 6:**
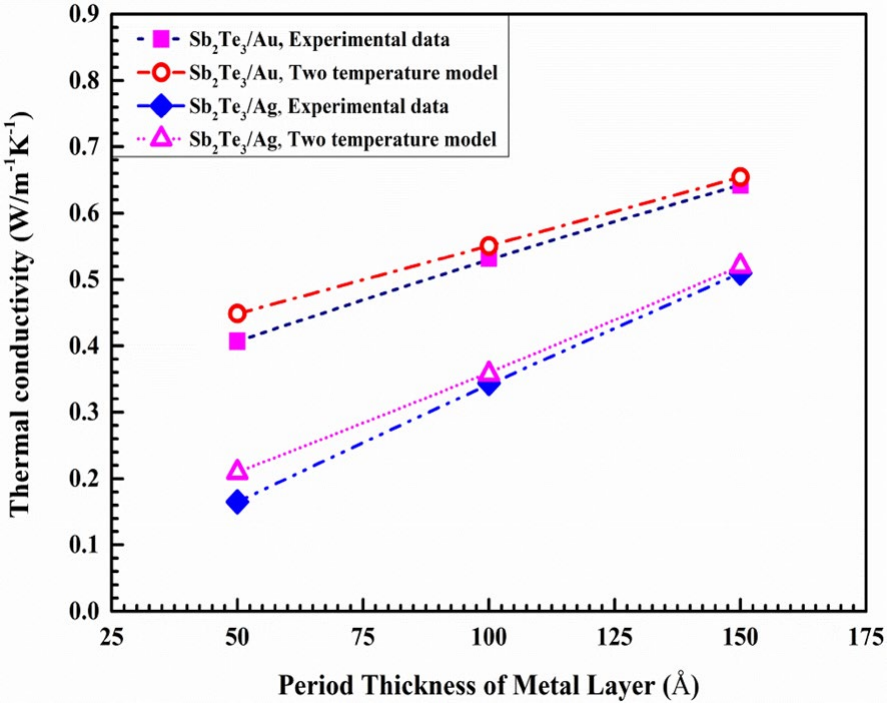
Simulation versus experimental results

In this simulation, electron thermal conductivity *k*_*e*_ and phonon thermal conductivity *k*_*p*_ for Ag at a temperature of 300 K were chosen as 370 and 4 Wm^−1^K^−1^, respectively. Electron and phonon thermal conductivity for Au at a temperature of 300 K were chosen as 314 and 2 Wm^−1^K^−1^ according to the references [46–49]. Electron–phonon coupling factor *G* was calculated according to Lombard et al. [50]. The phonon–phonon interfacial thermal resistance *R*_*i*_ was evaluated according to a simple acoustic mismatch model [51]. In order to study the impact of electron–phonon coupling on the thermal conductivity of metal–semiconductor multilayer systems, the cross-plane thermal conductivities of the periodic multilayer films for various materials (Sb_2_Te_3_/Au, Sb_2_Te_3_/Ag) were investigated using the two-temperature model. The obtained results are shown in Fig. 6.

It can be seen from Fig. 6 that the modeling results agree well with the measured data. The results indicate that the electron–phonon coupling mechanism plays an important role in determining the interfacial thermal resistance and that the effective thermal conductivities are affected by the nonequilibrium process between the electrons and phonons on the metal side. Based on analysis of the model, it can be found that the coupling factor G at the Sb_2_Te_3_-metal interface is in the range 10^16^–10^17^ W/m^3^K. Therefore, by choosing a proper period thickness of the metal layer, which changes the coupling length appropriately, the thermal conductivity of the metal-semiconductor multilayer thin film could be further reduced.

### Cross-plane thermal conductivities of Sb_2_Te_3_/Au multilayer thin films at various temperatures

**Fig. 7:**
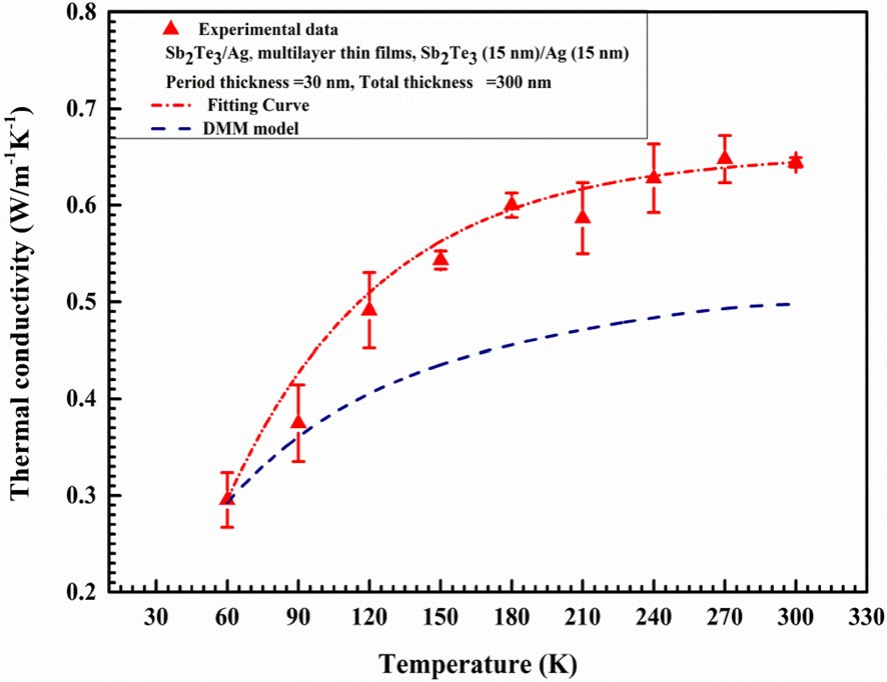
Cross-plane thermal conductivity of Sb_2_Te_3_/Ag as a function of temperature

In order to investigate the effects of temperature on the cross-plane thermal conductivities of the multilayer thin film samples, we measured the cross-plane thermal conductivity of the Sb_2_Te_3_/Ag multilayer thin film samples at various temperatures, and the obtained temperature-dependent cross-plane thermal conductivity data for the Sb_2_Te_3_/Ag multilayer thin film sample are summarized in Fig. 7.

It can be seen from Fig. 7 that the cross-plane thermal conductivity of the Sb_2_Te_3_/Ag multilayer thin film exhibited a lower value at room temperature (about 0.61 Wm^−1^K^−1^). In addition, the cross-plane thermal conductivity of the Sb_2_Te_3_/Ag multilayer thin film showed an increasing tendency with increasing temperature from 60 to 300 K. The observed ultralow thermal conductivity for the Sb_2_Te_3_/Ag multilayer thin film can be attributed to the high interfacial thermal resistance at the interface between Sb_2_Te_3_ and Ag. Based on the aforementioned analysis of the microstructure of the interface, a simple analytical model for thermal conduction was carried out. In order to elucidate the microscopic mechanism and compare with the experimental data at various temperatures, the diffusive mismatch model was employed. According to the diffuse scattering assumption, the net heat flow and thermal boundary conductance from material 1 to 2 can be written as

13 $$q_{net,DMM} = \frac{{k_{b}^{4} }}{{8\pi^{2} \hbar^{3} }}\left\{ {T_{1}^{4} \sum\limits_{j} {c_{1,j} \int_{0}^{{\frac{\hbar \omega }{{k_{b} T_{1} }}}} {\frac{{\alpha_{1 \to 2} \cdot z^{3} }}{{e^{z} - 1}}dz - T_{2}^{4} \sum\limits_{j} {c_{2,j} \int_{0}^{{\frac{\hbar \omega }{{k_{b} T_{2} }}}} {\frac{{\alpha_{2 \to 1} \cdot z^{3} }}{{e^{z} - 1}}dz} } } } } \right\}$$

14 $$h_{1 \to 2} = \frac{{\partial q_{net,DMM} }}{\partial T} = \frac{1}{4}\sum\limits_{j = 1}^{3} {\int_{\omega } {c_{1,j} \left( \omega \right)} } \alpha_{1 \to 2} \left( \omega \right)\hbar \omega Dos_{1} \left( \omega \right)\frac{{\partial f_{0} }}{\partial T}d\omega$$

where *c*_1,*j*_ and *c*_2,*j*_ are the group velocities of mode j, ℏ is Planck’s constant, *Dos*(*ω*) is the phonon density of states, and *f*_0_ is the Bose−Einstein distribution function. *α*_1→2_ is the transmission probability, and can be simplified as

15 $$\alpha_{1 \to 2} \left( \omega \right) = \frac{{\sum\limits_{j} {c_{2,j} \left( \omega \right)Dos_{2} \left( \omega \right)\delta_{{\omega ,\omega^{\prime}}} } }}{{\sum\nolimits_{j} {c_{1,j} \left( \omega \right)Dos_{1} \left( \omega \right)\delta_{{\omega ,\omega^{\prime}}} + \sum\nolimits_{j} {c_{2,j} \left( \omega \right)Dos_{2} \left( \omega \right)\delta_{{\omega ,\omega^{\prime}}} } } }}$$

For simplified calculations, a linear Debye approximation of the phonon dispersion relation has been employed. After obtaining the interfacial thermal conductance, we could model the total thermal resistance; finally, the effective thermal conductivity could be simply evaluated according to *k*_*e*_ = (*d*_1_ + *d*_2_)/2(*R*_1→2_). The obtained results were compared with the experimental data, as shown in Fig. 7. Of special note, a big assumption has been made in the present model in that the material structure is perfectly crystalline. However, the Sb_2_Te_3_/Ag multilayer thin film is polycrystalline or amorphous structure. Here, there are fairly large discrepancies between the experimental data and the predicted model results, even though the model provides better understanding of heat transport for the multilayer thin films at various temperatures.

## Conclusions

In this study, the cross-plane thermal conductivities of nanostructured Sb_2_Te_3_/(Cu, Ag, Au, Pt) thermoelectric multilayer thin film systems were investigated. The effects of interface microstructure on the thermal conductivity of the multilayer thin film were examined. In addition, the temperature profile was also investigated. The experimental results indicated that nanometer multilayer interface microstructures have a significant effect on the cross-plane thermal conductivity. The nanometer interface microstructure could influence phonon scattering process. In addition, the experimental and model results suggested that, for Sb_2_Te_3_/metal (Cu, Ag, Au, and Pt) thermoelectric multilayer thin films, the electron–phonon coupling that occurred at the interface also influenced the cross-plane thermal conductivity of multilayer thin film. By optimizing the material system properly, the cross-plane thermal conductivities of thermoelectric multilayer thin films can be further reduced without deteriorating the electron transfer properties. However, the correlation between microstructure and thermal conductivity still needs to be explored. More important, the thermal transport mechanisms at the atomic scale need to be revealed. In addition, experimental study indicated that the cross-plane thermal conductivity of semiconductor/metal thermoelectric multilayer thin film increased with period thickness of the metal layer, which suggested that electron thermal conductivity contributed a certain proportion to total thermal conductivity. For the Sb_2_Te_3_/metal (Cu, Ag, Au, Pt) multilayer thin films, interface effects and electron–phonon coupling effects dominated its thermal transport properties. In conclusion, cross-plane thermal conductivity reduction by engineering interface conditions may offer a promising route to improve thermoelectric conversion efficiency.
